# Studying the Physical and Chemical Properties of Polydimethylsiloxane Matrix Reinforced by Nanostructured TiO_2_ Supported on Mesoporous Silica

**DOI:** 10.3390/polym15010081

**Published:** 2022-12-25

**Authors:** Sari Katz, Noa Lachman, Nir Hafif, Lilach Rosh, Alexander Pevzner, Amir Lybman, Tal Amitay-Rosen, Ido Nir, Hadar Rotter

**Affiliations:** 1 Department of Space Environment, Soreq NRC, Yavne 81800, Israel; 2 Department of Materials Science and Engineering, Tel Aviv University, Tel Aviv 6997801, Israel; 3 Department of Physical Chemistry, Israel Institute for Biological Research, Ness Ziona 74100, Israel

**Keywords:** polydimethylsiloxane-based composite, reactive protective barrier, mechanical properties, thermal properties, ordered mesoporous silica, TiO_2_ nanoparticles

## Abstract

In this study, a reactive adsorbent filler was integrated into a polymeric matrix as a novel reactive protective barrier without undermining its mechanical, thermal, and chemical properties. For this purpose, newly synthesized TiO_2_/MCM/polydimethylsiloxane (PDMS) composites were prepared, and their various properties were thoroughly studied. The filler, TiO_2_/MCM, is based on a (45 wt%) TiO_2_ nanoparticle catalyst inside the pores of ordered mesoporous silica, MCM-41, which combines a high adsorption capacity and catalytic capability. This study shows that the incorporation of TiO_2_/MCM significantly enhances the composite’s Young’s modulus in terms of tensile strength, as an optimal measurement of 1.6 MPa was obtained, compared with that of 0.8 MPa of pristine PDMS. The composites also showed a higher thermal stability, a reduction in the coefficient of thermal expansion (from 290 to 110 ppm/°C), a 25% reduction in the change in the normalized specific heat capacity, and an increase in the thermal degradation temperatures. The chemical stability in organic environments was improved, as toluene swelling decreased by 40% and the contact angle increased by ~15°. The enhanced properties of the novel synthesized TiO_2_/MCM/PDMS composite can be used in various applications where a high adsorption capacity and catalytic/photocatalytic activity are required, such as in protective equipment, microfluidic applications, and chemical sensor devices.

## 1. Introduction

Polydimethylsiloxane (PDMS) is an organosilicon elastomer with a silicon–oxygen backbone of a [-Si (CH_3_)_2_O-] repeated unit, also known as silicone rubber [1,2]. PDMS’s valuable properties include hydrophobicity due to the methyl side groups, transparency, gas permeability, flexibility, biocompatibility, high strain-to-failure, and the ability to restore its original shape when stress is removed, in addition to being easily fabricated and having a low cost [2]. Furthermore, it can be sealed to itself and other materials without any adhesive when it is plasma-oxidized [3]. PDMS has many applications in various fields. It can be used for protective coatings against corrosion, ice-retarding, anti-biofouling and fire retardant, [4] in micropump and microvalve systems [5,6,7], as membranes [8], as wearable stretchable fabric for sensing and physical protection [9,10,11], and for piezoelectric usage [12]. Nevertheless, PDMS has some drawbacks that limit its application as a structural material. It has a low Young’s modulus and it is susceptible to organic solvents, which cause swelling and the deformation of its structural configuration. Moreover, PDMS has a high coefficient of thermal expansion (CTE) (i.e., it has a dimensional instability to temperature changes and is a highly hydrophobic material with low surface energy). To overcome PDMS’s disadvantages and to adjust it to its various application fields, much research has been carried out to study the effects of adding fillers to PDMS, such as carbon nanotubes, metal oxides, fumed silica, carbon black, carbon fibers, montmorillonite, etc., on its mechanical [13,14,15], physical [16], chemical [2,17], and optical [18,19,20] properties. Among the various fillers, silica has been broadly used in a large number of commercial PDMS products, such as sealants, adhesives, membranes, and elastomers. The silica serves as a reinforcing agent that improves the mechanical properties of the PDMS matrix due to its inherently weak intermolecular force [21]. Reinforcing silicone rubber with mesoporous silica nanoparticles significantly improved its mechanical and thermal stability [22,23,24,25] as compared to a nonporous silica reinforcement. The mesoporous silica reinforcement was more effective at reducing the CTE values because its robust silica framework suppressed the thermal expansion of the silicone rubber confined in the mesopores. The silica framework also reduced the thermal mass loss and swelling and effectively increased the tensile strength and Young’s modulus.

Our previous study [11] demonstrated the ability to improve the permeation resistance of the PDMS matrix against hazardous chemical materials with a catalyst filler acting as a novel reactive protective barrier. The filler was based on a high-loading (45 wt%) TiO_2_ nanoparticle catalyst inside the pores of the ordered mesoporous silica, MCM-41. The new TiO_2_/MCM/PDMS composite combined the high adsorption capacity of MCM-41 with the chemical warfare agents’ (CWAs’) self-decontamination reactivity of TiO_2_. The incorporation of the TiO_2_/MCM catalyst into the inert PDMS matrix improved its protection capabilities by increasing the breakthrough time of the adsorbed vapors and reduced the environmental risk by minimizing the desorption of toxic vapors. Those features are especially relevant for the key structural components of personal protective equipment (PPE) that have short breakthrough times, such as PDMS. Using this composite in applications such as PPE and barriers requires high strength, flexibility, and chemical and thermal stabilities. Therefore, it is crucial to evaluate whether the incorporation of active adsorbents into the PDMS elastomeric polymer would damage its properties and functionality.

In the current research, a study of the mechanical, thermal, and dimensional stability behavior of PDMS filled with various TiO_2_/MCM loadings was carried out. The combination of TiO_2_ nanoparticles inside a mesoporous MCM-41-supported structure that is embedded into the PDMS matrix as a mechanical reinforcement filler has never been explored. The nanoparticles of TiO_2_, when used as a reinforcement, have a low volume fraction and limited dispersion due to nanoparticle agglomeration [26]. However, using TiO_2_/MCM as a possible reinforcement enables a higher volume fraction and the uniform dispersion of a TiO_2_ catalyst, thus improving the applicability of TiO_2_/MCM/PDMS.

## 2. Experimental Section

### 2.1. Chemicals

Mesostructured MCM-41-type silica (hexagonal) and titanium (IV) butoxide (Ti[n-BuO]_4_ (97% reagent grade) were used in the preparation of the additive. Hexane (analytical grade) was used as a solvent. Polydimethylsiloxane (PDMS) was prepared from a SYLGARD 184 Silicone Elastomer Kit from Dow Corning Co (Midland, MI, USA). All chemicals were purchased from Sigma-Aldrich (St. Louis, MO, USA).

### 2.2. TiO_2_/MCM/PDMS Preparation

Thick films (~0.5 mm thickness) of a pristine PDMS matrix, as well as composite PDMS films embedded with TiO_2_/MCM at the different weight percentages of 2.5, 5, 10, and 15 wt%, were prepared.

First, MCM-41 mesoporous silica was modified by inducing a high loading (45 wt%) of nanostructured titanium dioxide (TiO_2_) particles via the controlled hydrolysis of titanium butoxide and condensation within the pores of MCM-41. The preparation procedure of the TiO_2_/MCM catalysts is described in detail in our previous work [11].

Pristine PDMS and composite films were prepared using a SYLGARD^TM^ 184 Silicone Elastomer Kit. For example, in order to prepare a composite film with a 10 wt% of TiO_2_/MCM, 0.22 g of the additive powder was sonicated in 2.5 mL of hexane for 2 h. The suspension was mixed with the PDMS precursors (2 g of the monomer and 0.2 g of the curing agent) and was poured into a polystyrene 6-well plate (9.61 cm^2^ area) that had been degassed in a desiccator for 1 h to eliminate trapped air bubbles. Curing and drying were performed along the following temperature ramp: 30 °C for 1 h, 50 °C for 1 h, 70 °C for 1 h, and 85 °C for 3 h. At least three replicates were tested for each sheet. The obtained sheet thickness was 0.5 mm ± 0.01 mm. For comparison, pristine PDMS films with the same dimensions were also prepared.

Hereinafter, the pristine PDMS film is denoted as PDMS and the PDMS films embedded with different loadings of TiO_2_/MCM-41 are denoted as 2.5, 5, 10, and 15 wt% of TiO_2_/MCM/PDMS.

### 2.3. Characterization Methods

The chemical structure and composition of the samples were measured using attenuated total reflectance Fourier transform infrared (ATR–FTIR) and Raman spectroscopy. The infrared analysis was carried out with an ATR diamond crystal accessory using an INVENIO S FTIR spectrometer (Bruker, Bremen, Germany) equipped with a DGTS detector. FTIR spectra were recorded within the wavenumber range from 4000 cm^−1^ to 400 cm^−1^ at a 4 cm^−1^ resolution. The Raman spectra were recorded on a Malvern Morphologi 4-ID microscope (Malvern, UK) using a 785 nm wavelength excitation laser. The spectra were measured with a 1800 lines/mm grating monochromator with a spectral resolution of 4 cm^−1^. The objective magnification was ×5 with an exposure time of 20 s under low-power conditions. Raman spectral analysis was carried out by creating a baseline. The baseline correction used asymmetric least squares smoothing to remove the effects of fluorescence, photoemission, photoluminescence, and other additive features in the spectra. The spectra were then normalized by dividing the baseline spectra by the 1414 cm^−1^ PDMS band, which removed uncertainties in regard to the laser-intensity fluctuations. Scanning electron microscopy (SEM) and energy dispersive spectroscopy (EDS) were performed by using the Phenom ProX desktop SEM. Samples’ images were obtained at 15 kV under low-vacuum conditions. The mapping of elements (Si, O, C, Ti) was carried out via cross-sectional imaging of the thick-film samples. The samples’ mechanical properties were measured using the TA Instruments DMA Q800 in tension mode. For each material, a set of 5 film samples with dimensions of ~0.5 mm × 2.5 mm × 20 mm were placed under a load control of 5 N/min at room temperature, and the displacement response was measured. Young’s modulus was calculated from the slope of the stress–strain curve up to a 40% elongation to ensure analysis consistency for the samples’ elastic region. The toughness was calculated based on the area under the stress–strain curve. The thermal properties of the samples were measured via differential scanning calorimetry (DSC) on a DSC823e from Mettler Toledo (Greifensee, Switzerland). The samples (~20 mg) went through three thermal cycles. During the first cycle, the samples were heated from −150 ℃ to 100 ℃ at a rate of 10 ℃/min, followed by 5 min of isothermal holding. During the second cycle, the samples were cooled from 100 ℃ to −150 ℃ at the same rate. Throughout the last cycle, the samples were heated in a similar manner as the first cycle. The glass transition temperatures (T_g_) were measured for all samples during the last heating cycle. The normalized specific heat capacity (∆Cp_n_) is calculated as follows [27]:

(1) $$\Delta {Cp}_{n}=\frac{\Delta Cp}{\left(1-x\right)}$$

where ∆Cp is the change in the specific heat capacity during the glass transition and x is the mass fraction of the adsorbent.

In order to measure the CTE, film samples with the dimensions of ~0.5 mm × 2.5 mm × 20 mm were placed in the TA Instruments DMA Q800 under a tensile loading of 0.1 N (about a 0.08 MPa stress level), and heated from room temperature to 150 ℃ at a rate of 5 ℃/min. The samples’ CTE was calculated based on the displacement-temperature slope in the temperature region of 45–70℃. Thermogravimetric analysis (TGA) was carried out on an STA6000 from PerkinElmer (Waltham, MA, USA). The samples (40 mg) were first equilibrated for 5 min at 30 °C, and then heated to 800 °C at a heating rate of 10 °C/min under a nitrogen atmosphere (60 mL/min). Peak degradation temperatures were found using the first-derivative curves.

Solvent swelling measurements were carried out by immersing pristine and composite PDMS samples (~0.5 mm × 5 mm × 20 mm) in a sealed vial that contained 5 mL of aqueous solution or toluene at room temperature for 3 h, until equilibrium was reached. The samples were wiped and weighed. The mass swelling ratio (R_m_) was calculated based on the mass changes before and after swelling.

The sessile droplet method was used to measure the contact angle for each sample via the DSA 25drop shape analyzer from KRUSS. For each measurement, a 5 μL volume of either toluene or distilled water was placed on the surface. The procedure was repeated at least 5 times and the averaged values were reported. The optical properties of the film samples were observed through a UV-2401 PC spectrophotometer (Shimadzu, Kyoto, Japan) equipped with the ISR-2200 integrating sphere model. Spectra were recorded at room temperature between 200 and 800 nm by using a halogen lamp with a range of 400–800 nm and a deuterium lamp with a range of 160–400nm. The sample thickness was 0.5 mm.

## 3. Results and Discussion

Nanosized TiO_2_ particles (~4 nm) were produced inside the pores of MCM-41 at a high loading of 45 wt% while preserving the texture and structure of the support. The surface area (520 m^2^/g) and pore volume (0.26 cm^3^/g) of the reactive adsorbent remained sufficiently high for adsorption and catalysis capabilities. The structural and textural properties of the TiO_2_/MCM adsorbent have been described in detail in our previous work [11].

### 3.1. Chemical Structure and Composition

**Figure 1:**
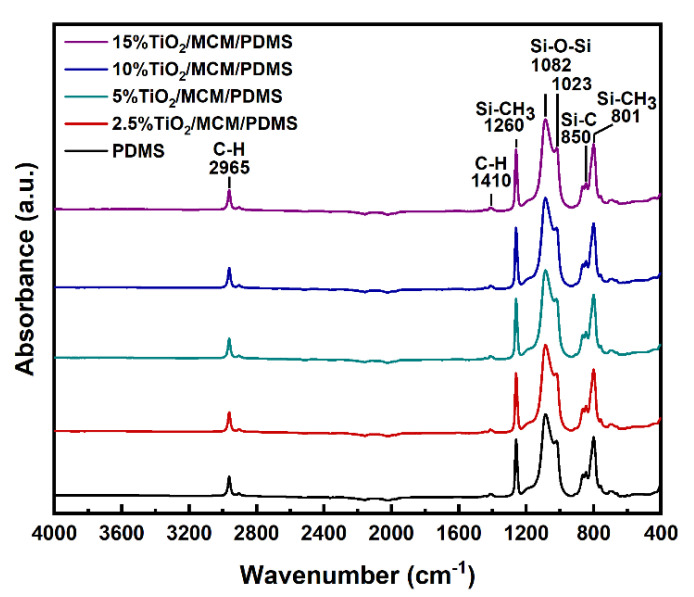
FTIR spectra of pristine PDMS and TiO_2_/MCM/PDMS composites.

ATR–FTIR spectroscopy was used to study the formation of bonds in the pristine PDMS and in the composite materials (Figure 1). The FTIR of all the samples showed excellent agreement with the previous FTIR results for PDMS [18,28,29,30,31,32,33]. No additional peaks were observed for the composite materials, suggesting that no chemical bonds were formed between the adsorbent and the PDMS matrix [34]. A peak at ~2140 cm^−1^, corresponding to Si-H stretching, was not observed for any sample, as shown in Figure 1, verifying that a hydrosilylation crosslinking reaction occurred and that a similar high crosslink density was achieved for all samples [31,32]. The peaks at 2965 cm^−1^ correspond to C-H stretching, the peaks at 1410 cm^−1^ correspond to -C-H bending, and the peaks at 1260 cm^−1^ correspond to the -CH_3_ deformation in Si-CH_3_. The observed peaks at 1023 and 1082 cm^−1^ are attributed to Si-O-Si flexing and stretching vibrations, respectively. Si-C bands and rocking peaks for Si-CH_3_ were observed in the 825–865 and 785–815 cm^−1^ regions, respectively [33]. There is a higher absorption below 700 cm^−1^, compared to in the pristine PDMS, due to the broad peaks at ~660 cm^−1^ that correspond to Ti-O-Ti stretching in TiO_2_ anatase crystals [35].

**Figure 2:**
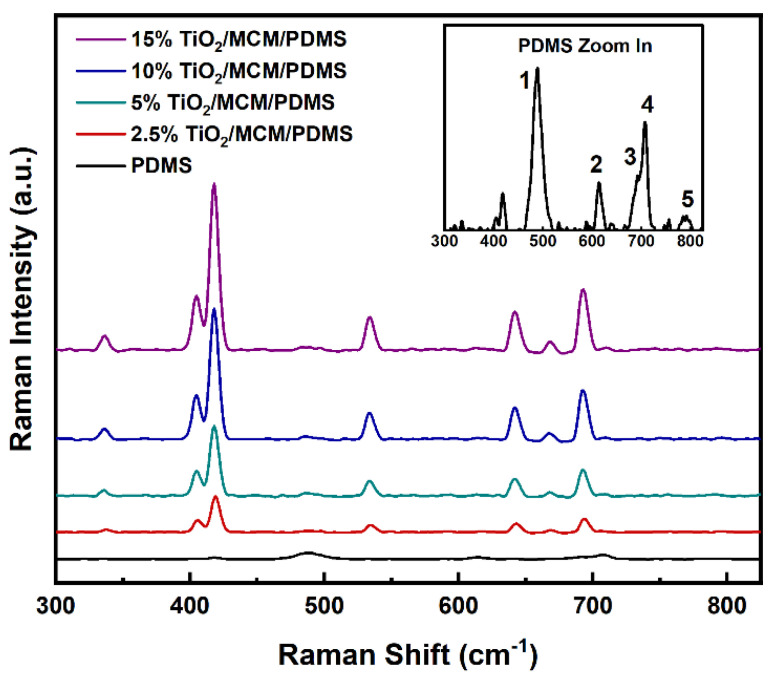
Raman spectra of pristine PDMS and TiO_2_/MCM/PDMS composites.

Raman spectroscopy was used as a complementary method for the study of the chemical structure (Figure 2). The Raman vibration modes of the pristine PDMS were found to be 488, 614, 687, and 707cm^−1^, as seen in Figure 2 for numbers 1, 2, 3, and 4, respectively. These vibrations were also found in previously published data [19,36,37], and they correspond to Si-O-Si symmetric stretching, Si-CH_3_ symmetric rocking, Si-CH_3_ symmetric rocking, and Si-C symmetric stretching. The vibration band at 787 cm^−1^ (number 5 in Figure 2) is attributed to both CH_3_ asymmetric rocking and Si-C asymmetric stretching. With the addition of TiO_2_/MCM, higher intensity levels of the 405, 418, and 687 cm^−1^ bands are found for oxides [38], and the appearance of new vibration bands at 336 and 642 cm^−1^ can be attributed to the Ti-O anatase [39,40]. The 534 cm^−1^ band is assigned to the delocalized vibrational modes arising from nonbridging oxygen in the silica network [41].

### 3.2. Mechanical Properties of the TiO_2_/MCM/PDMS Composite Materials

**Figure 3:**
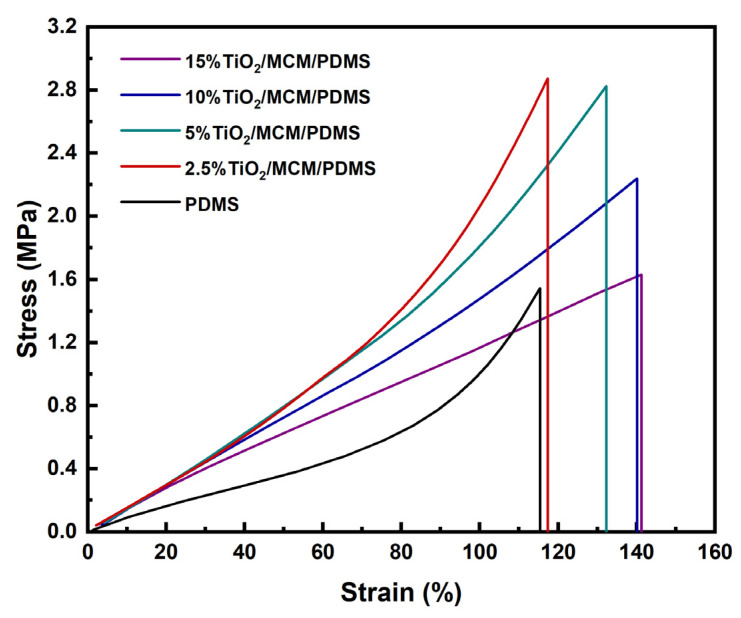
The stress–strain curves of TiO_2_/MCM/PDMS composites at various TiO_2_/MCM wt%.

The stress–strain curves for all TiO_2_/MCM/PDMS composites were measured using the DMA under a controlled load. A representative example for each material is presented in Figure 3.

The tensile stress–strain curves show a higher elongation at break with the addition of a filler, and a maximum value of 150 MPa for the toughness of 10 wt% TiO_2_/MCM reactive adsorbent was obtained, as compared to a value of 62 MPa for the pristine PDMS. The toughness further decreases as the TiO_2_/MCM content increases from 10 wt% to 15 wt%. The characteristic nonlinear strain-hardening effect disappears in the plastic region of the composite after the addition of a >5 wt% filler. This effect is due to the filler–matrix interactions limiting the chains’ movements.

**Figure 4:**
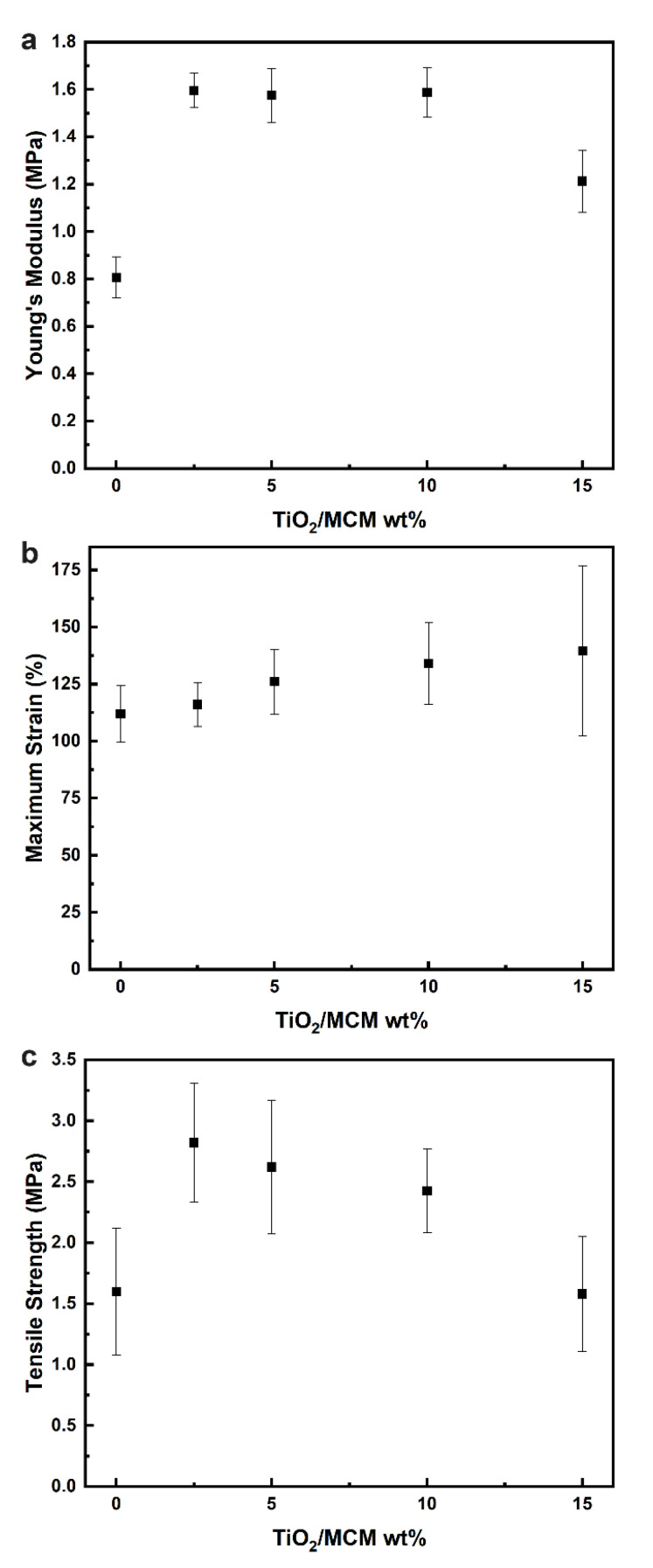
The elastic modulus (**a**), maximum strain (**b**), and tensile strength (**c**) of TiO_2_/MCM/PDMS composites at various TiO_2_/MCM wt%.

Based on the results obtained from the tensile testing of the composite materials, the mechanical properties were evaluated, including the Young’s modulus (Figure 4a), maximum strain (Figure 4b), and tensile strength (Figure 4c). For the pristine PDMS, the Young’s modulus, tensile strength, and maximum strain are 0.81 MPa, 1.54 MPa, and 115.39%, respectively. Pure PDMS usually has a Young’s modulus between 1.32 and 2.97 MPa, and a tensile strength from 3.51 to 5.13 MPa. These differences can be explained by the curing conditions (humidity, curing agent ratio, temperature, etc.), which have been shown to have a significant effect on the final mechanical, thermal, and physical properties of PDMS [42]. With the incorporation of TiO_2_/MCM, the mechanical properties were enhanced significantly, as all samples had a higher elastic modulus, tensile strength, maximum strain, and toughness compared to the pristine PDMS. Incorporation of the 10 wt% TiO_2_/MCM into the PDMS polymer produced a maximum strain with the highest improvement of 134% without undermining the maximum Young’s modulus (1.6 MPa), which was doubled compared to that of the pristine PDMS, and significantly increased the tensile strength of the material (2.4 MPa). Above a 10 wt% of TiO_2_/MCM, Young’s modulus and the tensile strength were reduced. Increasing the adsorbent concentration resulted in more contact sites and higher interfacial interactions between the polymer chains and the TiO_2_/MCM particles. Consequently, the stiffness and the strength of the composites were increased due to the improved stress transfer from the PDMS matrix to the adsorbent [43]. The decrease in tensile properties above a 10 wt% is due to the formation of bigger aggregates that reduce the dispersion of the filler in the PDMS matrix, leading to a reduction in stress transferring efficiency. This finding is supported by the SEM/EDS images of the TiO_2_/MCM/PDMS composites at various TiO_2_/MCM wt% and by Ti element mapping (see Supplementary Materials, Figure S1). Figure S1 shows that the TiO_2_/MCM particles were dispersed uniformly in the PDMS matrix. As the loading increased above 10 wt%, larger aggregates at a higher concentration were formed, which reduced the dispersion, and can explain the higher standard deviation.

### 3.3. Thermal Properties

**Figure 5:**
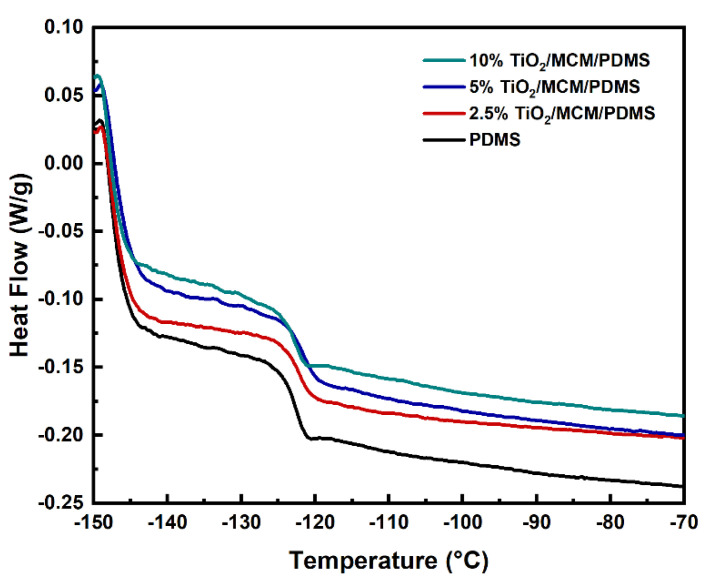
DSC curves of PDMS composites at various TiO_2_/MCM wt%.

Glass transition temperatures of the PDMS composites with different loadings of adsorbents were evaluated via DSC. Figure 5 shows the DSC curves during the second heating cycle. Pristine PDMS showed a T_g_ of −123 °C (Table 1); similar results were also found in ref. [30]. The addition of the catalytic adsorbent (TiO_2_/MCM) slightly increased the T_g_ (≤2 ℃), as can be viewed in Figure 5 and Table 1, indicating that the T_g_ is not significantly affected by the filler as reported previously [43,44]. This phenomena is due to the fact that the glass transition is only affected by the fraction of the unmodified polymer outside the interfacial layer [45].

The reduction in the mobility of the PDMS composites is reflected by the decrease in the normalized specific heat capacity, which was pronounced with the addition of TiO_2_/MCM (Table 1). ∆Cp_n_ (Equation (1)) relies on a proportion of the polymer participating in the glass transition and is relative to the degree of freedom of segmental motion [46,47]. As presented in Table 1, the values of ∆Cp_n_ for TiO_2_/MCM/PDMS composites are all lower than those for pristine PDMS. This reduction seems to be related to the polymer–adsorbent interfacial interactions that create an immobilized polymer layer on the surface of the adsorbent [27,46].

The samples’ CTE was determined from the measured displacement-temperature curves under tensile loading. All samples showed a relatively constant CTE value between 45 ℃ and 70 ℃; hence, this region was used to calculate the CTE. The CTE of the PDMS matrix was found to be 290 ppm/℃, which was slightly lower than the value (310 ppm/℃) reported by Dow for the SYLGARD™ 184 [48]. This difference can be explained by the change in curing conditions (time, temperature, etc.), which have a significant effect on the properties of PDMS, as reported by other studies [34,42]. The CTE values that were obtained for the TiO_2_/MCM/PDMS composites with a 2.5, 5, and 10 wt% adsorbents were 110, 110, and 150 ppm/℃, respectively, which were 48–62% lower than that of the pristine PDMS. Thus, the addition of TiO_2_/MCM induces a higher thermal stability of the material. The new reactive mesoporous adsorbent, which has a low CTE of silica-ceramic materials, introduced its higher dimensional stability when above room temperature (45–70 °C) to the final modified PDMS [25]. According to the literature [23,24], some PDMS chains may penetrate the pores of MCM-41 due to capillary forces. As a result, their thermal expansion will be limited by the framework of MCM-41.

**Figure 6:**
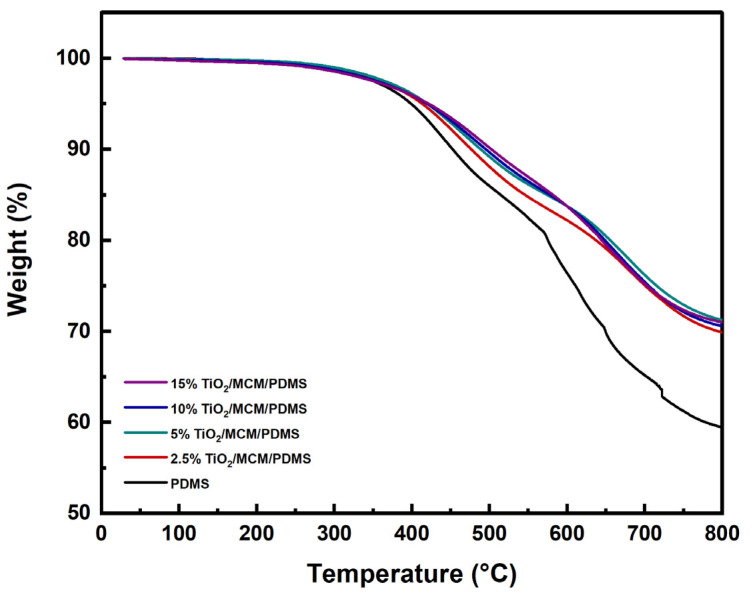
TGA thermograms of PDMS and TiO_2_/MCM/PDMS composites.

The thermal stability of the TiO_2_/MCM/PDMS composites at high temperatures was evaluated via the thermogravimetric analysis, which measured the sample’s weight loss as a function of the temperature. Several characteristic parameters were used to analyze the thermal stability and degradation process, such as: the temperature for a 5% weight loss (T_5_), the temperature for a 10% weight loss (T_10_), and the temperature at which the maximum rate of mass loss is observed (T_max_), in addition to the char residues [43,49]. The TGA thermograms of the pristine PDMS and PDMS composites are shown in Figure 6. The major weight loss of the pristine PDMS and PDMS composites occur in the temperature range of 400–690 °C, with two main degradation steps. The first step is around 450 °C, which is due to the decomposition of hydrocarbon species, such as methane and ethylene, from the vinyl terminal sites of PDMS, as well as the decomposition of Si-C_2_H_3_. The second step is around 650 °C, which is due to the decomposition of silicon-based oligomers and depolymerization [30,49]. It appears that the incorporation of TiO_2_/MCM, as a function of adsorbent loading, increased the thermal stability parameters of T_5_, T_10_, and T_max_ of the PDMS composites (Table 2). The initial decomposition temperature, T_5_, increased with the addition of the adsorbent, rising from 399 °C for the pristine PDMS to 422 °C for the 15 wt% TiO_2_/MCM/PDMS composite. The T_10_ change was more pronounced, and the temperature increased from 453 °C for the pristine PDMS to 502 °C for the 15 wt% TiO_2_/MCM/PDMS sample. The T_max_ for the pristine PDMS was found to be 628°C, whereas the T_max_ of the 2.5–15 wt% TiO_2_/MCM/PDMS was 676–682 °C. Thus, introducing the TiO_2_/MCM ceramic particles to the PDMS improves the thermal stability of the polymer. Furthermore, the char residues of the PDMS composites (69.9–71.2%) were higher than the char residue of the pristine PDMS (59.5%). This can be considered as an additional indication that adding TiO_2_/MCM improves the PDMS composites’ resistance to thermal degradation, but not in a linear manner. In summary, the thermal degradation results of the TiO_2_/MCM/PDMS composites are strengthened by the lower ΔCp_n_ and CTE results which were presented earlier.

### 3.4. Chemical Stability

Solvent swelling tests were used to study the effect of the adsorbent filler on the PDMS’s chemical stability. The swelling of the TiO_2_/MCM/PDMS composites was measured in the organic solvent toluene, as a hydrophobic representative material, and in water as a hydrophilic, physiologic representative material. The mass swelling ratio (R_m_) is calculated based on the mass change as follows:

(2) $$R_{m}=\frac{m-m_{0}}{m_{0}}*100$$

where m_0_ and m symbolize the weights of the PDMS before and after absorbing toluene and water, respectively.

**Figure 7:**
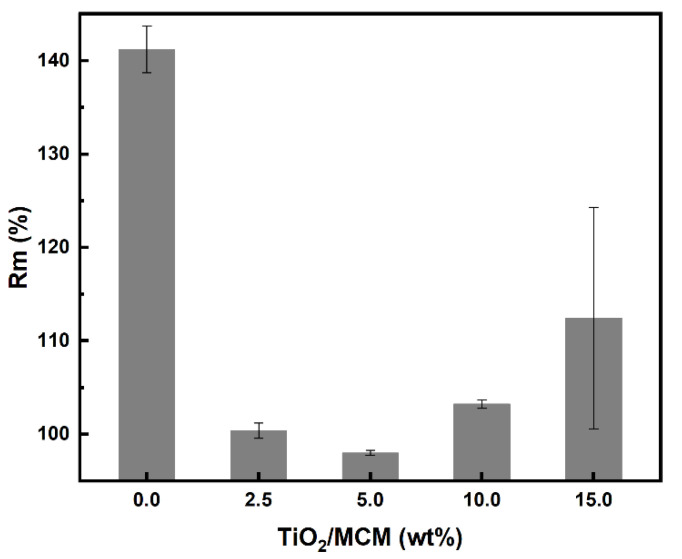
Toluene swelling ratio, (R_m_) %, of PDMS and TiO_2_/MCM/PDMS composites.

With regard to water absorption, the swelling ratio results were negligible, which indicates that there is no effect due to the incorporation of TiO_2_/MCM adsorbents in the PDMS matrix. The hydrophobicity of the PDMS was not impaired. On the other hand, for toluene absorption, the composite samples showed a significant decrease (about 40%) in the swelling ratio, as compared to the pristine PDMS (Figure 7).

In general, a decrease in the swelling ratio is associated with an increase in the crosslink density. However, as shown previously through the FTIR analysis, all samples, regardless of the TiO_2_/MCM concentration, reached a similar degree of crosslinking, implying that the adsorbent has a weak interaction with the PDMS polymer matrix. As suggested by Gupta et al. [30], the adsorbent reduces the free volume between the polymer molecules, and thus, partially inhibits the solvent from entering the matrix during swelling.

### 3.5. Surface Hydrophilicity/Hydrophobicity

**Figure 8:**
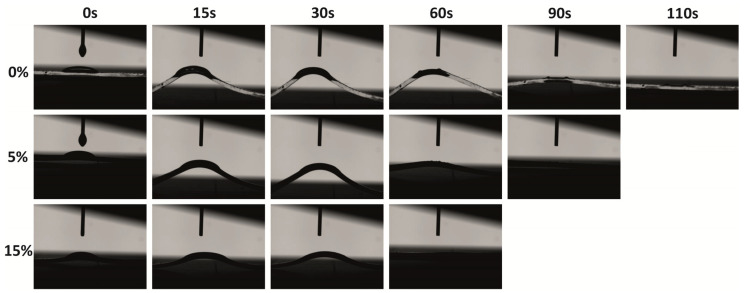
Toluene’s contact angle images for PDMS and TiO_2_/MCM/PDMS composites (at various TiO_2_/MCM wt%) as a function of time.

The surface hydrophilic/hydrophobic properties were evaluated via contact angle measurements using either toluene or water. As expected, the pristine PDMS matrix demonstrated hydrophobic surface properties, as the water contact angle was 100°, which is higher than 90°. Similar results were also achieved by Lamberti [19]. The addition of 2.5–15wt% TiO_2_/MCM slightly decreased the contact angle to the range of 85°–95° due to the hydrophilic nature of the TiO_2_/MCM additive as compared to the PDMS matrix. Since the incorporation of the TiO_2_/MCM adsorbent just slightly reduced the hydrophobic nature of the PDMS, it can still be used in, for example, microfluidic systems, where a uniform laminar flow of an aqueous solution without friction and deformation is required [50]. Furthermore, the hydrophobic nature of PDMS was depicted by the low contact angle of 29° when introduced to toluene. The addition of 2.5–15 wt% TiO_2_/MCM increased the contact angle to the range of 37°–49°, yielding a surface that is less hydrophobic and more stable against organic solvent swelling as compared to pristine PDMS. Figure 8 demonstrates the images of the contact angle as a function of time for the pristine PDMS, 5 wt% TiO_2_/MCM/PDMS composite, and 15 wt% TiO_2_/MCM/PDMS composite. It can be noticed that the addition of a reactive adsorbent reduced the deformation curvature, as well as the relaxation time (from 110 s to 60 s), i.e., the PDMS composites are less deformable and can quickly recover their original shape. In cases where exposure to an organic substance is required, absorption and deformation of the structure may occur, and therefore, TiO_2_/MCM/PDMS composites are beneficial.

### 3.6. Optical Properties

**Figure 9:**
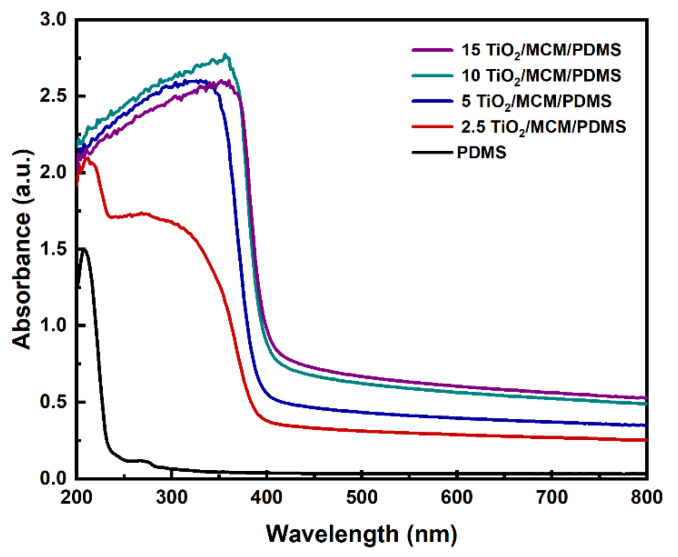
UV–Vis absorbance spectra of PDMS and TiO_2_/MCM/PDMS composites.

The effects of the incorporation of adsorbent particles on the optical properties of PDMS were studied. The ultraviolet–visible (UV–Vis) spectra of PDMS and the 2.5, 5, 10, and 15 wt% TiO_2_/MCM/PDMS composites are shown in Figure 9. The pristine PDMS was relatively transparent with a transmittance above 92% in the range from 350 to 800 nm. As the loading increased from 2.5 to 15 wt%, the transmittance of the PDMS composites decreased to be in the range of 27%-54%, as can be viewed due to the higher absorbance in the visible range. The TiO_2_/MCM particles can cause light scattering, which decreases the transmittance of PDMS gradually as the particle concentration increases [25]. In the UV range, pristine PDMS has a negligible absorbance between 250–400 nm. With the addition of TiO_2_/MCM, the absorbance increased significantly and had a clear cutoff at 400 nm, which was due to the optical properties of the TiO_2_ anatase crystalline structure that absorb in the UV range, but not in the visible range [51]. The addition of TiO_2_/MCM to PDMS enables both adsorption and photocatalytic activity. These features can be applied in applications for environmental pollutants’ treatment [52].

## 4. Conclusions

A novel reactive mesoporous adsorbent material, i.e., TiO_2_/MCM, was synthesized and embedded inside a PDMS matrix as a potential protective barrier due to its capabilities of adsorption and the catalytic degradation of hazardous materials. The mechanical, thermal, optical, and chemical stability properties of TiO_2_/MCM/PDMS composites at different weight percentages (2.5–15 wt%) were studied. The composite materials demonstrated superior mechanical (Young’s modulus, tensile strength, and maximum strain) and thermal (CTE, ΔCp, and T_max_) properties compared to the pristine PDMS matrix. Furthermore, the TiO_2_/MCM addition increased the dimensional stability in a hydrophobic environment, as the swelling was reduced by 40% and the relaxation time was decayed (from 110 s to 60 s). Regarding the optical properties, the adsorbent addition reduced transparency as a function of composite loading. However, incorporating TiO_2_/MCM enables UV absorption, thus inducing the photocatalytic ability of the PDMS composite. The results indicate that the 10 wt% TiO_2_/MCM addition to the PDMS matrix is optimal, due to the combined effects of the adsorption/catalytic reactivity and mechanical/thermal/optical/chemical stability properties. Such reinforcements can also be applied in other applications where the combined effects of TiO_2_ nanoparticles and MCM as a reactive adsorbent are favorable, such as organic/biologic pollutants’ degradation of wastewater, chemical sensor devices, coatings against biofouling in microfluidic devices, and coatings against corrosion.

## Figures and Tables

**Table 1:** Thermal properties of TiO_2_/MCM/PDMS composites.

TiO_2_/MCM/PDMS (wt%)	T_g_ (℃)	ΔCp_n_ (J/gK)	CTE (ppm/°C)
0	−123.1	0.305	290
2.5	−122.3	0.232	110
5	−122.0	0.244	110
10	−123.2	0.249	150

**Table 2:** Thermal data of TiO_2_/MCM/PDMS composites.

TiO_2_/MCM/PDMS (wt%)	T_5_ (℃)	T_10_ (℃)	T_max_ (℃)	Char Residue (%)
0	399	453	628	59.5
2.5	414	476	676	69.9
5	420	488	679	71.2
10	420	494	681	70.6
15	422	502	682	71.0

## Data Availability

Data is contained within the article or Supplementary Material.

## Supplementary Materials

The following supporting information can be downloaded at https://www.mdpi.com/article/10.3390/polym15010081/s1: Figure S1: Back scattered electrons (BSE) images of cross-sectional TiO_2_/MCM/PDMS composites (left column) and elements mapping by EDS at various adsorbent wt%.

## References

[1] Waldbaur A., Rapp H., Länge K., Rapp B.E. (2011). Let There Be Chip—Towards Rapid Prototyping of Microfluidic Devices: One-Step Manufacturing Processes. Anal. Methods.

[2] Ariati R., Sales F., Souza A., Lima R.A., Ribeiro J. (2021). Polydimethylsiloxane Composites Characterization and Its Applications: A Review. Polymers.

[3] Mukhopadhyay R. (2007). When PDMS Isn’t the Best. Anal. Chem..

[4] Eduok U., Faye O., Szpunar J. (2017). Recent Developments and Applications of Protective Silicone Coatings: A Review of PDMS Functional Materials. Prog. Org. Coat..

[5] RajM K., Chakraborty S. (2020). PDMS Microfluidics: A Mini Review. J. Appl. Polym. Sci..

[6] Johnston I.D., Tracey M.C., Davis J.B., Tan C.K.L. (2005). Micro Throttle Pump Employing Displacement Amplification in an Elastomeric Substrate. J. Micromechanics Microengineering.

[7] Wu X., Kim S.H., Ji C.H., Allen M.G. (2011). A Solid Hydraulically Amplified Piezoelectric Microvalve. J. Micromechanics Microengineering.

[8] Scholes C.A., Stevens G.W., Kentish S.E. (2012). Membrane Gas Separation Applications in Natural Gas Processing. Fuel.

[9] Victor A., Ribeiro J., Araújo F.S. (2019). Study of PDMS Characterization and Its Applications in Biomedicine: A Review. J. Mech. Eng. Biomech..

[10] Ramji R., Khan N.T., Muñoz-Rojas A., Miller-Jensen K. (2015). “Pop-Slide” Patterning: Rapid Fabrication of Microstructured PDMS Gasket Slides for Biological Applications. RSC Adv..

[11] Rotter H., Osovsky R., Hafif N., Pevzner A., Nir I. (2022). Nanostructured TiO_2_/MCM-41-Functionalized PDMS as a Reactive Protective Barrier against Chemical Warfare Agents via Adsorption and Catalyzed Degradation. Ind. Eng. Chem. Res..

[12] Kumar V., Kumar A., Han S.S., Park S.-S. (2021). RTV Silicone Rubber Composites Reinforced with Carbon Nanotubes, Titanium-Di-Oxide and Their Hybrid: Mechanical and Piezoelectric Actuation Performance. Nano Mater. Sci..

[13] Wu C.-L., Lin H.-C., Hsu J.-S., Yip M.-C., Fang W. (2009). Static and Dynamic Mechanical Properties of Polydimethylsiloxane/Carbon Nanotube Nanocomposites. Thin Solid Film..

[14] Burns G.T., Taylor R.B., Xu Y., Zangvil A., Zank G.A. (1992). High-Temperature Chemistry of the Conversion of Siloxanes to Silicon Carbide. Chem. Mater..

[15] Zhao W., Li T., Li Y., O’Brien D.J., Terrones M., Wei B., Suhr J., Lucas Lu X. (2018). Mechanical Properties of Nanocomposites Reinforced by Carbon Nanotube Sponges. J. Mater..

[16] Vlassov S., Oras S., Timusk M., Zadin V., Tiirats T., Sosnin I.M., Lõhmus R., Linarts A., Kyritsakis A., Dorogin L.M. (2022). Thermal, Mechanical, and Acoustic Properties of Polydimethylsiloxane Filled with Hollow Glass Microspheres. Materials.

[17] Tavares M.T.S., Santos A.S.F., Santos I.M.G., Silva M.R.S., Bomio M.R.D., Longo E., Paskocimas C.A., Motta F.V. (2014). TiO_2_/PDMS Nanocomposites for Use on Self-Cleaning Surfaces. Surf. Coat. Technol..

[18] Dalod A.R.M., Grendal O.G., Blichfeld A.B., Furtula V., Pérez J., Henriksen L., Grande T., Einarsrud M.A. (2017). Structure and Optical Properties of Titania-PDMS Hybrid Nanocomposites Prepared by In Situ Non-Aqueous Synthesis. Nanomaterials.

[19] Lamberti A. (2015). Microfluidic Photocatalytic Device Exploiting PDMS/TiO_2_ Nanocomposite. Appl. Surf. Sci..

[20] Silva V.P., Paschoalino M.P., Gonçalves M.C., Felisberti M.I., Jardim W.F., Yoshida I.V.P. (2009). Silicone Rubbers Filled with TiO_2_: Characterization and Photocatalytic Activity. Mater. Chem. Phys..

[21] Chen D., Liu Y., Zhang H., Zhou Y., Huang C., Xiong C. (2013). Influence of Polyhedral Oligomeric Silsesquioxanes (POSS) on Thermal and Mechanical Properties of Polydimethylsiloxane (PDMS) Composites Filled with Fumed Silica. J. Inorg. Organomet. Polym. Mater..

[22] Suzuki N., Kiba S., Kamachi Y., Miyamoto N., Yamauchi Y. (2012). Unusual Reinforcement of Silicone Rubber Compounds Containing Mesoporous Silica Particles as Inorganic Fillers. Phys. Chem. Chem. Phys..

[23] Suzuki N., Kiba S., Kamachi Y., Miyamoto N., Yamauchi Y. (2011). Mesoporous Silica as Smart Inorganic Filler: Preparation of Robust Silicone Rubber with Low Thermal Expansion Property. J. Mater. Chem..

[24] Suzuki N., Kamachi Y., Takai K., Kiba S., Sakka Y., Miyamoto N., Yamauchi Y. (2014). Effective Use of Mesoporous Silica Filler: Comparative Study on Thermal Stability and Transparency of Silicone Rubbers Loaded with Various Kinds of Silica Particles. Eur. J. Inorg. Chem..

[25] Liu J., Zong G., He L., Zhang Y., Liu C., Wang L. (2015). Effects of Fumed and Mesoporous Silica Nanoparticles on the Properties of Sylgard 184 Polydimethylsiloxane. Micromachines.

[26] Cui X., Zhu G., Pan Y., Shao Q., Zhao C., Dong M., Zhang Y., Guo Z. (2018). Polydimethylsiloxane-Titania Nanocomposite Coating: Fabrication and Corrosion Resistance. Polymer.

[27] Wang Z., Zhang H., Liu Q., Wang S., Yan S. (2022). Effect of 3-Mercaptopropyltriethoxysilane Modified Illite on the Reinforcement of SBR. Materials.

[28] Efimenko K., Wallace W.E., Genzer J. (2002). Surface Modification of Sylgard-184 Poly (Dimethyl Siloxane) Networks by Ultraviolet and Ultraviolet/Ozone Treatment. J. Colloid Interface Sci..

[29] Téllez L., Rubio J., Rubio F., Morales E., Oteo J.L. (2004). FT-IR Study of the Hydrolysis and Polymerization of Tetraethyl Orthosilicate and Polydimethyl Siloxane in the Presence of Tetrabutyl Orthotitanate. Spectrosc. Lett..

[30] Gupta N.S., Lee K.S., Labouriau A. (2021). Tuning Thermal and Mechanical Properties of Polydimethylsiloxane with Carbon Fibers. Polymers.

[31] Stafie N., Stamatialis D.F., Wessling M. (2005). Effect of PDMS Cross-Linking Degree on the Permeation Performance of PAN/PDMS Composite Nanofiltration Membranes. Sep. Purif. Technol..

[32] Berean K., Ou J.Z., Nour M., Latham K., McSweeney C., Paull D., Halim A., Kentish S., Doherty C.M., Hill A.J. (2014). The Effect of Crosslinking Temperature on the Permeability of PDMS Membranes: Evidence of Extraordinary CO_2_ and CH4 Gas Permeation. Sep. Purif. Technol..

[33] Nour M., Berean K., Griffin M.J., Matthews G.I., Bhaskaran M., Sriram S., Kalantar-zadeh K. (2012). Nanocomposite Carbon-PDMS Membranes for Gas Separation. Sens. Actuators B Chem..

[34] Konku-Asase Y., Yaya A., Kan-Dapaah K. (2020). Curing Temperature Effects on the Tensile Properties and Hardness of γ−Fe_2_O_3_ Reinforced PDMS Nanocomposites. Adv. Mater. Sci. Eng..

[35] Beghi M., Chiurlo P., Costa L., Palladino M., Pirini M.F. (1992). Structural Investigation of the Silica-Titania Gel/Glass Transition. J. Non Cryst. Solids.

[36] Cai D., Neyer A., Kuckuk R., Heise H.M. (2010). Raman, Mid-Infrared, near-Infrared and Ultraviolet–Visible Spectroscopy of PDMS Silicone Rubber for Characterization of Polymer Optical Waveguide Materials. J. Mol. Struct..

[37] Bae S.C., Lee H., Lin Z., Granick S. (2005). Chemical Imaging in a Surface Forces Apparatus: Confocal Raman Spectroscopy of Confined Poly(Dimethylsiloxane). Langmuir.

[38] Hadjiivanov K.I., Panayotov D.A., Mihaylov M.Y., Ivanova E.Z., Chakarova K.K., Andonova S.M., Drenchev N.L. (2021). Power of Infrared and Raman Spectroscopies to Characterize Metal-Organic Frameworks and Investigate Their Interaction with Guest Molecules. Chem. Rev..

[39] Kusabiraki K. (1986). Infrared Spectra of Vitreous Silica and Sodium Silicates Containing Titanium. J. Non Cryst. Solids.

[40] Ma W., Lu Z., Zhang M. (1998). Investigation of Structural Transformations in Nanophase Titanium Dioxide by Raman Spectroscopy. Appl. Phys. A Mater. Sci. Process..

[41] Scannell G., Barra S., Huang L. (2016). Structure and Properties of Na_2_O-TiO_2_-SiO_2_ Glasses: Role of Na and Ti on Modifying the Silica Network. J. Non Cryst. Solids.

[42] Johnston I.D., McCluskey D.K., Tan C.K.L., Tracey M.C. (2014). Mechanical Characterization of Bulk Sylgard 184 for Microfluidics and Microengineering. J. Micromechanics Microengineering.

[43] Anoop V., Sankaraiah S., Chakraborty S., Mary N.L. (2022). Enhanced Mechanical, Thermal and Adhesion Properties of Addition Cured Polydimethylsiloxane Nanocomposite Adhesives. Int. J. Adhes. Adhes..

[44] Bosq N., Guigo N., Persello J., Sbirrazzuoli N. (2014). Melt and Glass Crystallization of PDMS and PDMS Silica Nanocomposites. Phys. Chem. Chem. Phys..

[45] Fragiadakis D., Pissis P. (2007). Glass Transition and Segmental Dynamics in Poly (Dimethylsiloxane)/Silica Nanocomposites Studied by Various Techniques. J. Non Cryst. Solids.

[46] Klonos P., Panagopoulou A., Bokobza L., Kyritsis A., Peoglos V., Pissis P. (2010). Comparative Studies on Effects of Silica and Titania Nanoparticles on Crystallization and Complex Segmental Dynamics in Poly (Dimethylsiloxane). Polymer.

[47] Bershtein V.A., Egorova L.M., Yakushev P.N., Pissis P., Sysel P., Brozova L. (2002). Molecular Dynamics in Nanostructured Polyimide-Silica Hybrid Materials and Their Thermal Stability. J. Polym. Sci. Part B Polym. Phys..

[48] Corporation. D.C. Information about Dow Corning Silicone Encapsulants. https://krayden.com/pdf/dow_silicone_encapsulant.pdf.

[49] Venkatachalam S., Hourlier D. (2019). Heat Treatment of Commercial Polydimethylsiloxane PDMS Precursors: Part I. Towards Conversion of Patternable Soft Gels into Hard Ceramics. Ceram. Int..

[50] Heo B., Fiola M., Yang J.H., Koh A. (2020). A Low-Cost, Composite Collagen-PDMS Material for Extended Fluid Retention in the Skin-Interfaced Microfluidic Devices. Colloid Interface Sci. Commun..

[51] Gao X., Wachs I.E. (1999). Titania–Silica as Catalysts: Molecular Structural Characteristics and Physico-Chemical Properties. Catal. Today.

[52] Hickman R., Walker E., Chowdhury S. (2018). TiO_2_-PDMS Composite Sponge for Adsorption and Solar Mediated Photodegradation of Dye Pollutants. J. Water Process Eng..

