# Supplementary material for: Studying the Physical and Chemical Properties of Polydimethylsiloxane Matrix Reinforced by Nanostructured TiO2 Supported on Mesoporous Silica
Source: Polymers (Basel). 2022 Dec 25;15(1):81. doi: 10.3390/polym15010081 (PMC9824354; doi:10.3390/polym15010081)
Supplement: Supplementary file 1 [file polymers-15-00081-s001.zip › polymers-2115022-supplementary.pdf]

Supplementary Materials

Studying the Physical and Chemical Properties of Polydimethylsiloxane Matrix Reinforced by Nanostructured TiO_2 Supported on Mesoporous Silica

Sari Katz [1,*], Noa Lachman [2], Nir Hafif [3], Lilach Rosh [3], Alexander Pevzner [3], Amir Lybman [3], Tal Amitay-Rosen [3], Ido Nir [3] and Hadar Rotter [3,*]

[1] Department of Space Environment, Soreq NRC, Yavne 81800, Israel
[2] Department of Materials Science and Engineering, Tel Aviv University, Tel Aviv 6997801, Israel
[3] Department of Physical Chemistry, Israel Institute for Biological Research, Ness Ziona 74100, Israel
* Correspondence: sarik@soreq.gov.il (S.K.); hadarr@iibr.gov.il (H.R.)

SEM/EDS images of TiO_2/MCM/PDMS composites at various TiO_2/MCM wt% and elements mapping

SEM/EDS images of the TiO_2/MCM/PDMS composites at various TiO_2/MCM wt% and C, Si, O, Ti elements mapping (Figure S1) show that the TiO_2/MCM particles were dispersed uniformly in the PDMS matrix. As the loading increased above 10 wt% bigger aggregates at higher concentration were formed.

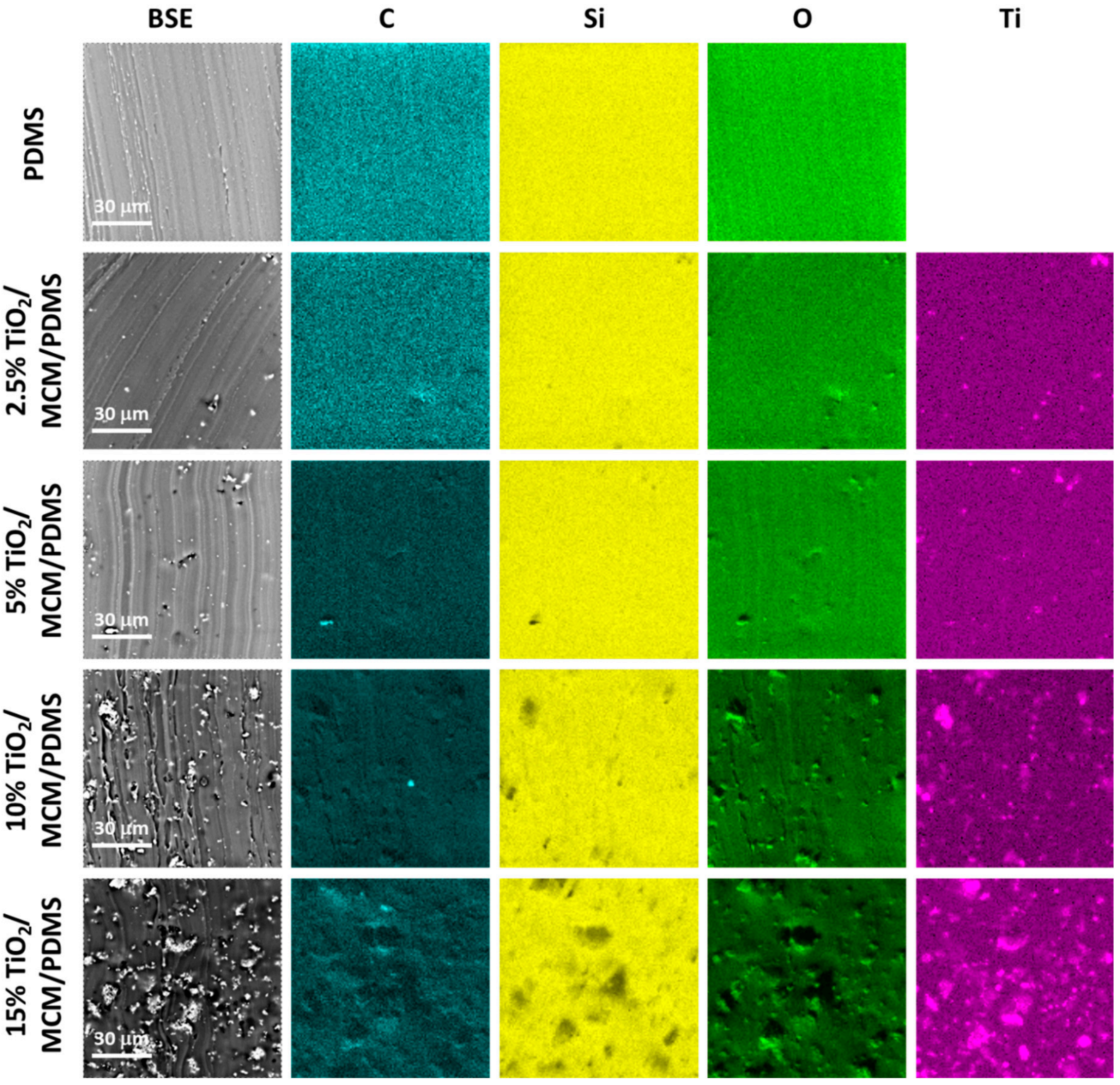

Figure S1. Back scattered electrons (BSE) images of cross-sectional TiO_2/MCM/PDMS composites (left column) and elements mapping by EDS at various adsorbent wt%.
